# The challenges of entering the metaverse: An experiment on the effect of extended reality on workload

**DOI:** 10.1007/s10796-022-10244-x

**Published:** 2022-02-12

**Authors:** Nannan Xi, Juan Chen, Filipe Gama, Marc Riar, Juho Hamari

**Affiliations:** 1 Faculty of Information Technology and Communication Sciences, Tampere University, Kalevantie 4, 33100 Tampere, Finland; 2 School of Technology and Innovations, University of Vaasa, Wolffintie 34, 65200 Vaasa, Finland; 3 School of Business Administration, Anhui University of Finance and Economics, Benghu, 233030 China; 4 Chair of Information and Communication Management, Technical University of Berlin, Straße des 17. Juni 135, 10623 Berlin, Germany

**Keywords:** Mixed reality, Virtual reality, Augmented reality, Metaverse, Workload, NASA Task Load Index

## Abstract

Information technologies exist to enable us to either do things we have not done before or do familiar things more efficiently. Metaverse (i.e. extended reality: XR) enables novel forms of engrossing telepresence, but it also may make mundate tasks more effortless. Such technologies increasingly facilitate our work, education, healthcare, consumption and entertainment; however, at the same time, metaverse bring a host of challenges. Therefore, we pose the question whether XR technologies, specifically Augmented Reality (AR) and Virtual Reality (VR), either increase or decrease the difficulties of carrying out everyday tasks. In the current study we conducted a 2 (AR: with vs. without) × 2 (VR: with vs. without) between-subject experiment where participants faced a shopping-related task (including navigating, movement, hand-interaction, information processing, information searching, storing, decision making, and simple calculation) to examine a proposed series of hypotheses. The NASA Task Load Index (NASA-TLX) was used to measure subjective workload when using an XR-mediated information system including six sub-dimensions of *frustration, performance, effort, physical, mental, and temporal demand*. The findings indicate that AR was significantly associated with overall workload, especially mental demand and effort, while VR had no significant effect on any workload sub-dimensions. There was a significant interaction effect between AR and VR on physical demand, effort, and overall workload. The results imply that the resources and cost of operating XR-mediated realities are different and higher than physical reality.

## Introduction

Extended reality (XR) technologies are some of the most prominent new developments in information systems, processing, and management (Kim & Hall, 2019; Rauschnabel, 2021; Xi & Hamari, 2020), and offers an umbrella term that covers all forms of virtual and augmented reality technologies, which is often used interchangeably with the term mixed reality (Fast-Berglund et al., 2018; Kwok & Koh, 2021). Among XR-related literature, Augmented Reality (AR) and Virtual Reality (VR) have increasingly been investigated in various areas (Zhang et al., 2017; Pfeiffer et al., 2020; Manis & Choi, 2019; Yim et al., 2017; Klinker et al., 2020) showcasing the impact of this information systems development on a variety of fields. For example, XR has been used to increase the learning and working efficiency of students and employees by providing real-time information (Bednar & Welch, 2020; Lal et al., 2021; Lee, 2012) in education and training, as well as being considered as a way of improving the efficiency of physical rehabilitation (Afanasiev et al., 2018) in health information systems. In addition, in business, applications such as virtual try-on technologies (Kim & Forsythe, 2008), AR product catalog presentation (Poushneh & Vasquez-Parraga, 2017; Rese et al., 2014) and virtual reality shops (Peukert et al., 2019) provide opportunities for high-efficiency consumption and hedonic experiences. Especially under the global impact of the coronavirus pandemic, XR technologies are inheriting increasingly important roles in social and economic development. According to a report from Technavio (2020), the AR and VR market will accelerate at a compound annual growth rate of over 35% through 2020-2024 considering the impact of COVID-19. However, a significant number of people either anticipate or have determined based on first experiences that there are unsurmountable limitations and hurdles in using XR in terms of e.g., usability, comfort, mental effort, operation and physical interaction. Also, the cost and degree of user challenge is unclear when carrying out these types of virtually-assisted activities in XR-based information systems^1^.

In the literature, the challenges and difficulties of using information systems based on XR technologies have been interpreted as the workload that users would experience. The current understanding of the impacts of different XR technologies on workload is still in its infancy. There is a research gap in distinguishing the similarities and differences in the difficulties and costs of operating realities created by XR technologies (e.g., completing tasks and conducting activities). In addition, workload has a multidimensional nature and can be influenced by various factors, for example an individual’s motivation, past experience and ability, and the specific characteristics of tasks (Hart, 1982; Meshkati, 1988). A number of XR-related studies have examined the users’ workload when using specific devices (Caria et al., 2020; Wang et al., 2019) or methods (Barré et al., 2019; Jost et al., 2020), as opposed to assessing the efficiency and usability of XR technologies in completing tasks from a more general perspective. Furthermore, many studies have failed to observe whether these effects were due to the mediating technology, or to content that was intentionally added or modified. More importantly, granular research on how XR technologies affect different aspects of workload has been slow to emerge, and current literature is limited regarding research on specific dimensions of workload (see e.g., mental workload, Zhao et al., 2017; physical workload, Chihara & Seo, 2018; cognitive workload, Tremmel et al., 2019).

Thus, the *research objective* of this study is to develop an in-depth understanding of what kinds and how much workload users can experience when carrying out information processing tasks in XR environments. Accordingly, the current study design takes the form of a 2 (VR: with vs. without) × 2 (AR: with vs. without) between-subject experiment in a shopping context. A brick-and-mortar record store, as well as a 1-to-1 digital 3D “replica” of the store (using laser scanning and Unity 3D for modeling) were constructed as the experimental environments. The NASA Task Load Index (NASA-TLX) was applied as a subjective measure for evaluating six sub-dimensions of workload. The paper is structured as follows. The extant literature related to XR and workload is discussed for proposing hypotheses in the next section. The research method including participants, materials, measures and procedures is presented in section 2.1. Sections 2.2 and 2.3 present and discuss the results in terms of the effects of XR on overall workload and its sub-dimensions. The conclusion, research implications and practical implications are presented in section 2.3.1. Section 2.3.2 presents the limitations of the current study and future research directions.

## Background and hypotheses

### Workload

In the field of information systems, individual workload or the lack thereof is often operationalized simply as the general ease of use of an information system in a given task (e.g. ‘freedom from difficulty’ according to the technology acceptance model (TAM): Davis, 1989). While this conceptualization and construct has functioned as a workhorse in the bulk of the technology acceptance literature produced in the last decades (Lah et al., 2020), it provides a limited and one-dimensional understanding of workload and the usability of information systems. Specifically, it does not take into consideration the barriers that would prevent system adoption (see Taylor & Todd, 2001), and it does not reveal how such perceptions are formed (see Mathieson, 1991). In this study, we are interested in the usability of a multimodal information system when used in a rich information processing task. Therefore, this paper ventures to investigate more holistic conceptualizations and constructs of workload. One instrument that is widely used in similar situations where attention and cognitive processes are limited by the context and complexity of the task is the NASA-TLX instrument, which measures the perceived workload of an individual in a given information processing task that can include mental, physical, frustration-inducing, temporal, performance-related and effort-related workload dimensions. Therefore, beyond investigating a novel context of using information systems, this study also builds bridges between fields in the frontier between adoption and ease of use research, in relation to new technologies.

Workload is largely considered as the effort or cost (e.g., physical, mental, emotional) an individual devotes to accomplish a task (Hart, 2006; Hart & Wickens, 1990). It can be influenced by internal aspects such as an individual’s motivation or their past experience and ability, as well as external aspects such as the type, novelty, difficulty, and number of tasks that an individual completes (Hart, 1982; Meshkati, 1988). Originally rooted in attempts to measure the effort of flight-related tasks (see e.g., Li et al., 2020), the practice of assessing workload also became more and more relevant in contexts that transcend aviation. For example, due to the rapid technological advancements and the growing number of novel systems that aim at enhancing, among other factors, convenience, productivity and efficiency, it became increasingly relevant to scrutinize the workload demands of all sorts of information systems. Respectively, today workload is also used to evaluate the interface design of conventional computer systems and portable devices, including the technologies that support virtual and augmented vision (Hart, 2006). The goal often involves gaining a better understanding of how to design and improve systems so that the intended benefits are not compromised by excessive workload during their use. Generally speaking, ergonomists and designers of information technology are interested in creating technology in a way that reduces workload or at least keeps it within an acceptable range (e.g., Grier et al., 2008), as workload management is vital for user acceptance (Dang et al. 2020), productivity, performance, and user health (Jung & Jung, 2001; MacDonald, 2003). An important consideration is that users can only deal with a finite capacity of workload. Kantowitz (1987) describes the concept of spare capacity, which understands that as long as task demands are below an individual’s maximum workload capacity, then performance should not be impaired. However, with increasing task complexity or difficulty, the perceived workload intensifies and if the acceptable level is exceeded, performance will suffer. In the light of this rationale, it is not surprising that in the past decades, theories of task-technology-fit (Goodhue & Thompson 1995) and disciplines devoted to the usability of systems (e.g., Hoehle & Venkatesh, 2015; Lewis, 2014) have garnered great attention in the realm of human-computer-interaction.

The practical necessity to assess the workload involved in human-computer interaction brought forth a number of different evaluation approaches, including objective measures based on performance indicators and psychophysiological cues, as well as measures based on subjective experiences (e.g., Cain 2007; Tsang & Vidulich 2006). Whereas objective measures collect real-time performance data or measure physiological reactions (e.g., via electrodes), subjective measures rely on the self-assessment of the experienced workload by the subjects (Tsang & Vidulich, 2006). A general issue pertaining to the assessment of workload considers the circumstance that different tasks tend to be subject to different sources of workload (e.g., mental and physical), as well as the varying degrees to which each specific source is accountable for an individual’s perceived overall workload (i.e., weighted workload) (Hart & Staveland 1988). A weighting scheme aims at measuring workload more accurately, and requires users to evaluate the degree to which different dimensions of workload contribute to the overall workload of a specific task (Hart, 2006). One particular measurement that has been widely accepted and deemed functional to cover the multidimensional nature of workload and capable of accounting for the individual differences of humans with regard to their weighted perceptions of workload is the NASA Task Load Index (TLX) (Hart & Staveland, 1988). This index belongs to the class of subjective measurement instruments, and allows individuals to quantify their experienced workload via a weighted scheme and consists of six dimensions. These include (1) physical demand, (2) mental demand, (3) temporal demand, (4) performance, (5) effort, and (6) frustration. Table 1 specifies each dimension in more detail, based on the work by Hart (2006). What should be mentioned here is that a single effort scale (combining physical effort and mental effort) cannot capture the information needed to address the specific source of demands (Hart & Staveland, 1998). Thus, instead of asking subjects to introspect about the amount of mental or physical effort exerted, the NASA TLX instrument requires them to assess the objective physical and mental demands that are placed on them (Hart & Staveland, 1998).

**Table 1:** Explanation of Each Dimension of Workload in NASA-TLX Based on Hart (2006)

Sub-dimensions of workload	Explanation
Mental demand	Perceived mental and perceptual activity required by an individual to accomplish a given task (e.g., thinking, deciding, calculating, remembering, looking, searching).
Physical demand	Perceived physical activity required by an individual to accomplish a given task (e.g., pushing, pulling, turning, controlling, activating).
Temporal demand	Perceived time pressure due to rate or pace of the given task.
Effort	Perceived level of work (mental and physical) to realize performance level.
Performance	Perceived success in accomplishing the goals that are tied to the performed tasks.
Frustration	Perceived insecurity, discouragement, irritation, stress and annoyance versus perceived security, contentment, relaxation and complacency during task performance.

### Extended reality

Even though there seems to be a lack of consistency in the use of reality-related terms (e.g., Virtual Reality-VR, Augmented Reality-AR, Mixed Reality-MR, and Extended Reality-XR) in academic and professional fields (Flavián et al., 2019), AR and VR have been considered as the two core reality-virtuality technologies. With the development of multi-sensory technologies and modalities and the deepened conceptualized understanding of AR and VR, there is a consensus that any sensory experience can be augmented in a digital way (Harley et al., 2018) and also be virtualized (Boyd & Koles, 2019). In terms of AR, multimodal information such as smell, touch, taste and sound can be digitally overlaid on the current world (Azuma, 1997; Carmigniani et al., 2011; Riar et al., 2021) and AR users are not isolated from it (Rauschnabel, 2021). AR has been defined as the term for technologies for augmenting or altering the current reality (Riar et al., 2021), while in VR, all of the sensory information and stimulus of the ‘real reality’ is rather blocked and inhibited (Manis & Choi, 2019; Yim et al., 2017). Therefore, while VR has been considered as the digital technologies of choice for substituting the perceived reality (Xi & Hamari, 2021), AR and VR provide different kinds of experiences to users (Fromm et al., 2021). Regarding AR, the “augmenting” information and content can bring users interactivity, vividness and novelty (McLean & Wilson, 2019; Yim et al., 2017). Alternatively, VR has been believed to create immersiveness (Suh & Prophet, 2018), telepresence (Lee & Chung, 2008; Steuer, 1992), and the sense of “being there” (Heeter, 1992; IJsselsteijn & Riva, 2003). As a further extension, when combining AR and VR together, Augmented Virtuality (AV) can be constructed for a more hybrid experience. There are high expectations towards AR and VR on creating interesting, novel and playful experiences (Lin & Yeh, 2019; Raptis et al., 2018), however, an increasing number of studies have shown that the use of AR and VR in activities and completing tasks requires various resources and costs.

### Workload in extended realities

The current study assumes that both AR and VR technology will lead to a high overall workload compared with non-technology mediated reality, but will influence different sub-dimensions of workload. In the following, we describe how AR and VR are expected to influence the particular dimensions of workload, and derive them according to hypotheses.

#### Mental demand

On one hand, XR technologies have been found to offer perceptual and cognitive benefits such as supporting cognitive processing, mental elaboration and imagery, by providing visual cues that are lacking from the physical environment (e.g., Bogicevic et al., 2019; Fan et al., 2020; Heller et al., 2019; Park & Yoo, 2020), as well as by offering simulating environments and situations that would otherwise be costly or difficult to produce in the real world (e.g., Barré et al., 2019; Clifford et al., 2018). On the other hand, however, it seems that these benefits often come at the cost of additional mental effort. For example, previous studies point out that using AR can result in a higher cognitive load and mental strain (e.g., Dunleavy et al., 2009; Tarafdar et al., 2019; Weidinger et al., 2018). It seems that especially in AR, individuals may be confronted with a more challenging perceptual task in processing augmented reality simultaneously with the physical reality, as the different visual sources may demand more mental effort from individuals. According to multiple resource theory, the mental workload is affected by humans’ limited capacity to deal with several sources of attention simultaneously (Wickens, 2008). It is likely that in AR, individuals expend mental resources towards interpreting both reality-based and augmented reality-based spatial information (and possibly auditory or other multimodal information), while also having to deal with potential extraneous distractions.

In comparison, we can expect that in a pure VR setting, individuals only have to mentally process the virtual reality, and hence it should be similar to exclusively processing the perceived current reality. Individuals may perceive their surroundings through the VR interface as being natural, and therefore they do not have to exert additional cognitive effort (Wickens, 1992). Moreover, VR has the ability to immerse users into the mediated environment so that they feel “present” in the virtual world (Schuemie et al., 2001; Steuer, 1992). Through this sense of presence in the virtual environment, extraneous cognitive load and distractions may be blocked, and consequently, there is no divided attention between the real world and the virtual world. Ultimately, it seems that when compared to the perceived current reality and VR, individuals have face greater perceptual challenges in AR since the visual cues coming from multiple resources (i.e., the perceived current reality and the augmented reality) have to be combined. Thus, we expect that for AR, the level of mental demand will be higher compared to purely physical or VR environments, since in the latter conditions, individuals only have to process a single reality and are not confronted with various sources of multimodal information. This also implies that there will be no significantly different experiences in the mental workload between fully virtual reality (as well as the interaction of AR and VR) and the perceived current reality. Such assumptions are in line with prior studies which indicate that VR does not cause additional mental workload compared to purely physical situations (without VR) (e.g., Chao et al., 2017), and that AR entails more mental workload in comparison to situations without AR. Therefore, we propose the following hyphothesis:

- *H1:* a) AR has a positive effect, while b) VR has no effect on mental demand.

#### Physical demand

In addition to mental demand, prior studies postulate that using XR technologies can result in higher degrees of physical exertion (e.g., Chao et al., 2017; Dickinson et al., 2020; Madathil & Greenstein, 2017; Millais et al., 2018). This can mostly be explained by the fact that when individuals perform tasks in the perceived current reality, they are used to the involved movements and can execute them naturally and effortlessly, whereas in AR and VR, the same tasks often require more demanding physical effort because individuals have to use specific hardware and controls generally involve movement-based mechanisms. The physical workload is caused by movements such as pulling, pushing, pressing, reaching, holding, and so on (Hart et al., 1981), many of which are movement patterns required to control XR technologies. There seems to be ample support for the notion that XR can increase physical effort, with previous studies proposing that XR requires more vigorous interactions compared to other less interactive technologies, and that the controls such as movement, pointing and selecting can result in higher degrees of physical burden (Chao et al., 2017; Madathil & Greenstein, 2017; Millais et al., 2018). Given the above evidence, the second hypothesis states that:

- *H2:* a) AR has a positive effect and b) VR has a positive effect on physical demand.

#### Temporal demand

Temporal demand is related to perceived time pressure, which is the perception that there is an inadequate amount of time available to carry out the given tasks (Guo et al., 2020). Time pressure has been considered as the need for time allocation, i.e. allocating time between competing options (Guo et al., 2020). Prior knowledge on the psychology of time pressure underlines the influence of increased work intensity and multitasking (Szollos, 2009). We presume that by being exposed to multimodal features and also handling different realities at once, individuals may be more inclined to feel agitated and rushed, experiencing higher temporal demand through the intensified work demands. For example, users in augmented reality have to synchronously process information from different channels (e.g., digital information being displayed on an AR device and information coming from the physical environment). Therefore, in AR, users may experience a higher temporal demand compared to the non-AR enabled reality.

While VR users might feel stressed from unnaturally interacting with digital objects and the environment with devices while performing tasks, the perception of time pressure might be the same as in an environment without VR (e.g., the physical world). In the purely virtual world (see “magic circle”: Castronova, 2008), users have opportunities to conduct activities that are typically either not done well or safely in real life without concerns for the consequences involved (Schultheis & Rizzo, 2001). They can further have a higher self-efficacy to complete tasks (Shu et al., 2019) without following the rules, which reduces the pressure of time allocation. For instance, in some studies related to VR shopping tasks, users neither need to take virtual products back to shelves nor consider whether the product could be damaged without careful interaction. Therefore, they are more likely to feel that they have enough time to complete the given tasks. However, an “offset effect” might occur when considering the additional tasks brought on by manipulating in virtual reality due to the limitations of VR technology, and less task-related demands from “magic circle” nature of VR, which together lead to the same temporal demands as seen in environments without VR. Given the above, we propose that:

- *H3:* a) AR has a positive effect, while b) VR has no effect on temporal demand.

#### Effort, performance and frustration

Based on the assumption that XR can generate both higher mental and physical demands, and considering the notion that perceived effort is caused by the level of work (e.g., mental and physical) that an individual has to expend in order to reach a certain level of performance (Hart, 2006), it seems a valid assumption that engagement with XR will also necessitate greater effort compared to task performance in non-XR conditions. Moreover, compared to the smooth interactions that we can experience with physical environments and physical objects, the interaction with virtual environments and objects in XR can be perceived as being more strenuous and frustrating. Reasons for this may include technological insufficiencies such as an unsatisfactory mapping quality of virtual content (Poushneh, 2018), outages, or other malfunctions which can increase frustration (Ahsen et al., 2019). A lack of intuitiveness and deficiencies with the modalities for interacting with virtual objects can also lead to increased levels of frustration (Whitlock et al., 2018). Generally speaking, prior literature indicates that there can be certain detriments involved with using XR technologies, such as difficulties in controlling virtual objects as effortlessly as in a physical environment, as well as increased perceptions of stress, discomfort, disorientation, and especially in VR, the problem of cybersickness (Munafo et al., 2017; Sharples et al., 2008; Somrak et al., 2019; Weech et al., 2019). These impediments (both technological and in terms of the user experience) can increase the required effort and frustration levels of individuals when using XR technology. In line with the understanding of the effort and frustration dimensions stemming from workload theory which entail higher degrees of expended work to achieve performance levels, as well as higher levels of experienced stress, irritation, annoyance, and discouragement during task completion (Hart 2006), we hypothesize that both AR and VR can lead to higher perceptions of effort (H4a/H4b) as well as frustration (H6a/H6b).

It should be noted that this does not necessarily mean that individuals’ performance will suffer. Rather, users may have to expend more effort to achieve the same level of performance in XR compared to the physical reality. According to theories of workload, performance may stay consistent even if individuals have to expend higher mental or physical effort, as long as the task demands do not reach a critical limit (i.e., the maximum capacity of workload) (Grier et al., 2008; Kantowitz, 1987). Prior studies also indicate that XR can in fact increase performance (e.g., Chang & Hwang, 2018; Kim et al., 2018). However, this seems more likely if the XR environment provides some added value (e.g., in terms of extra information, content, better interaction, enhanced interface features, and a multimodal experience). If no such added value is provided, the nature of VR only substitutes the perceived current reality and the essence of AR only changes the way information is presented. Thus, by default, the augmentations do not provide any enhanced experience beyond what the physical reality has to offer, and it seems unlikely that performance gains can be achieved. In line with these arguments, we presume that there will be no significant difference in performance between executing tasks within XR and physical realities. Thus, we derive the following hypotheses:

- *H4:* a) AR has a positive effect and b) VR has a positive effect on effort.
- *H5:* a) AR has no effect, and b) VR has no effect on performance.
- *H6:* a) AR has a positive effect, and b) VR has a positive effect on frustration.

## Research method

### Research Design

To investigate how the six dimensions of workload differ in different extended realities, we conducted a 2 (VR: with vs. without) × 2 (AR: with vs. without) between-subject experiment with a psychometric survey (see Table 2). The shopping task featured is usually involved with a series of holistic mental, cognitive and behavioral costs such as information searching, processing, interactive and movement-based behavior, time pressure and decision making, and thus perfectly matches the goal of a workload-related study. Thus, a shopping scenario was designed as the experiment context and participants were required to complete a 10-minute task. Each participant was given a 10 euros gift card for buying products in the shop we designed. The level of compensation depended upon the products each participant selected (they could keep the selected products). The purpose of such a compensation scheme was to garner a realistic context of decision making in the shop as the compensation depended upon the rational choice of the participant. The study design and procedure adhered to the Finnish National Board on Research Integrity TENK Guidelines 2019.

**Table 2:** The 2 (VR: with vs. without)×2 (AR: with vs. without) Between-Subject Experiment Design

No.	*VR*	*AR*	Value*		Name	Reality	Information	Device
1	Without	Without	0	0	Non-XR shop	Physical	Traditional	N/A
2	Without	With	0	1	AR shop	Physical	Superimposed	HoloLens
3	With	Without	1	0	VR shop	Virtual	Traditional (digital)	Valve index & controllers
4	With	With	1	1	AV shop	Virtual	Superimposed	Valve index & controllers

* Operationalization as dummy variable for VR and AR

### Participants

From September to November 2019, a total of 165 student participants were recruited from a Finnish university to participate in a shopping-related experiment (see section 3.4*Procedure*). Participants were randomly assigned to one of four groups. Two participants were omitted due to their inability to use the VR headset to complete the experiment^2^ and one disqualified participant was also dropped. Table 3 provides the detailed demographic information of participants including gender, age, education, study area, income, and nationality. The final sample size was 157 (54.14% male, 56.05% 20-24 years old, 56.69% undergraduate students, and 56.69% less than 499 euros monthly income)^3^. The participants had diverse nationalities, coming from Finland, China, Germany, Vietnam, Spain, Russia, India, France, and elsewhere.

**Table 3:** Participant Characteristics (Mean and Standard Deviation)

Measure	Group 1 (*n* = 40)	Group 2 (*n* = 41)	Group 3 (*n* = 40)	Group 4 (*n* = 36)	*F*(3, 153)	*p*
Gender	67.50 %	46.34 %	50 %	52.78 %	1.396	.246
Age	3.58 (1.40)	3.24 (0.80)	3.33 (1.02)	3.11 (0.67)	1.426	.237
Education	1.63 (0.67)	1.44 (0.59)	1.55 (0.64)	1.36 (0.54)	1.388	.248
Income	1.87 (1.34)	1.68 (1.00)	2.05 (1.53)	1.32 (0.83)	2.220	.088
Height	5.93 (1.59)	5.51 (1.75)	5.30 (2.16)	5.47 (1.65)	0.859	.464
VR experience	1.98 (1.10)	1.95 (1.00)	2.10 (1.01)	1.61 (0.84)	1.651	.180

Gender was considered as a binary variable; the percentage of males in each group is presented in the table; Age was measured from 1 = less than 15 years old, 2 = 15-19, 3 = 20-24 to 11 = 60 or older; Education was measured from 1 = bachelor student, 2 = master student and 3 = PhD student; Income (pre-tax) was measured from 1 = 0-499 euros, 2 = 500-999 euros to 9 = 4000 euros or more; Height was measured from 1 = 150 cm or less, 2 = 151-155 cm, 3 = 156-160 cm, to 8 = Above 180 cm; VR experience was measured by the frequency of using VR devices, ranging from 1 = never, 4 = sometimes, to 7 = every day

### Materials

#### Shop

**Fig. 1:**
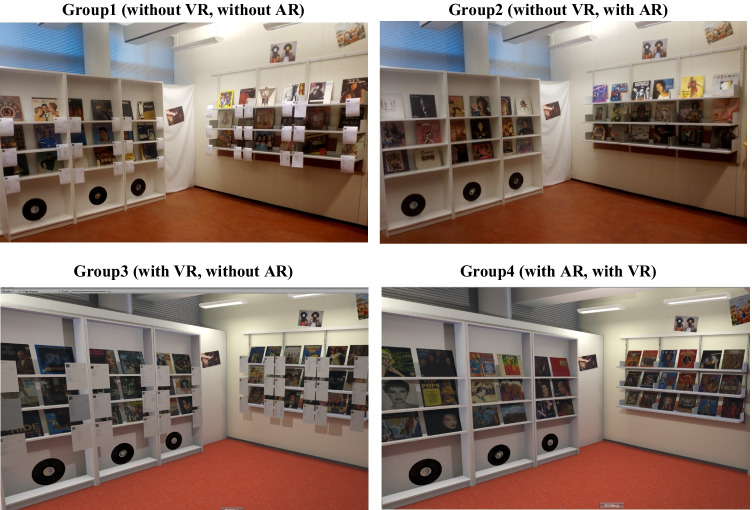
*Design of Four Shops in The Study. Note.* In the Group 2, Microsoft HoloLens 1 was used to present “pop-up” information; Valve Index VR headset was used in both Group 3 and 4. See open-access video link https://cutt.ly/XR-shopping

A physical LP record shop (4.24m×5.09m = 21.58m^2^) was built on the university campus, and functioned as the experimental setup for each condition. The shop floor plan, size, decoration and layout were identical in each condition. Fifty-four LP records were displayed on three different walls of the room, and each wall was equipped with three layers of shelves. For the Group 1 which is designed to control other groups, the shop functioned as a common style physical shop and the product information was presented on printed papers attached to the shelves. For Group 2, the shop functioned similarly except that the product information was displayed through an AR headset display (Microsoft HoloLens: see section 3.3*Apparatus*). For Group 3, the same room was used. The physical shop was scanned for 3D reconstruction by using an active scanning method, with a LiDAR sensor. Then the shop was modeled and refined in Blender with 1:1:1 scale and then imported to Unity. Thus, the shopping environment used in the “control” condition was reconstructed in virtual reality (e.g., textures, lighting conditions, geometry and “attached” product information). For Group 4, the environment combined the condition in Group 3 with the superimposed product information as in the condition of Group 2. See Fig. 1.

#### Product and its information

Purchasing second-hand English LP records (0.314m×0.314m, price categories of 3, 6 and 9 euros) was deemed as a suitable activity for the study as it represents a human activity that requires multimodal, multisensory interaction and effort. Moreover, students are the main consumers of music-related products. The average expenditure on a music hobby was 81.81 euros per year among the 157 participants in the current study. However, we wished to minimize any participant familiarity with specific artists/bands on display among the records in the shop. Therefore, all records in the shop were acquired second-hand and produced before the 1990s. To confirm our expectations of previous product knowledge, in the post-survey, we measured previous product knowledge by seven items based on 7-point Likert scale that were adapted from Awasthy et al. (2012), and the results indicate that the participants were not familiar with the products featured in the shop (*M* = 2.58, *SD* = 1.13). In addition, LP-record packaging is efficient to be modeled to match the physical versions of the products and the interactive quality for a computer-based environment. In the VR shop and AV shop, the front and back covers of the LP records were scanned in high quality and added as 3D objects into the shopping programs.

**Fig. 2:**
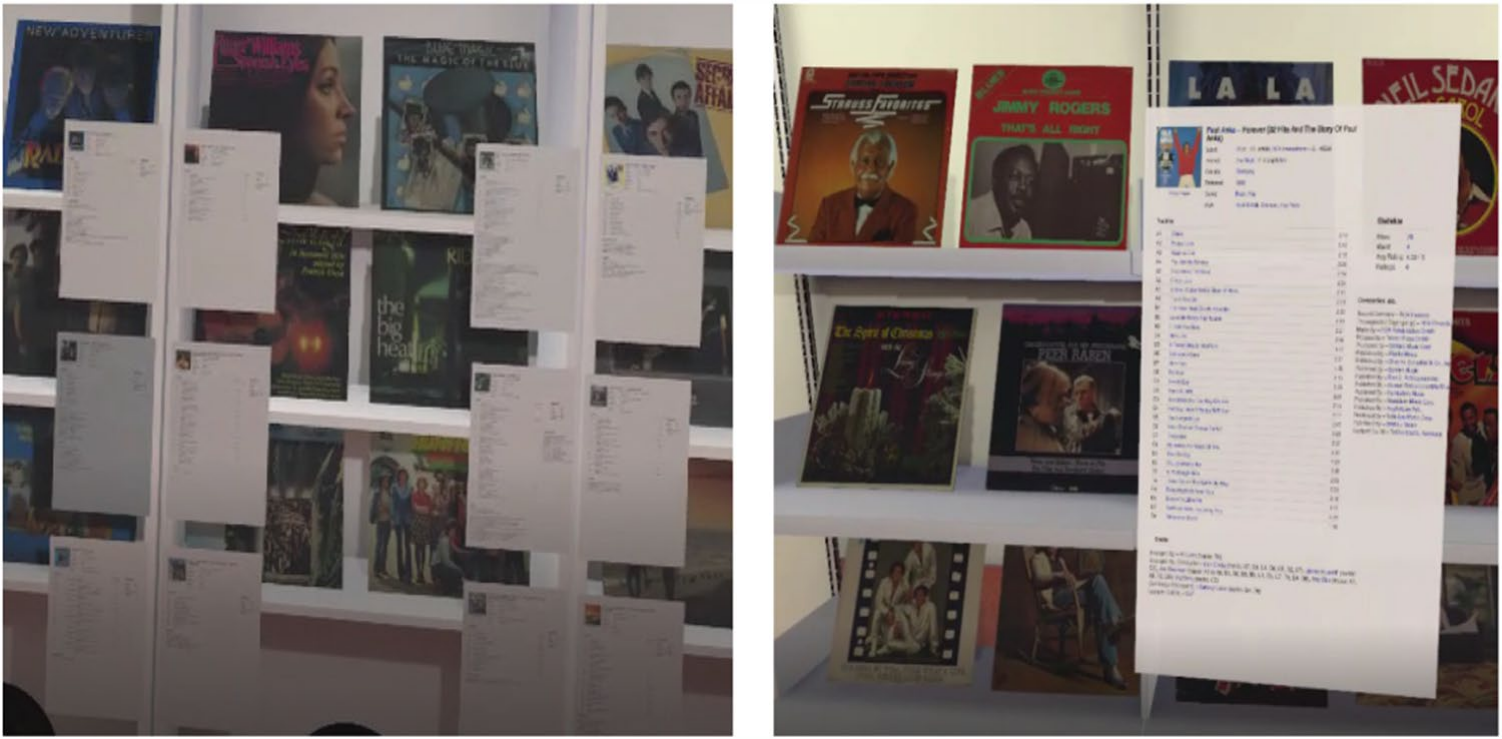
*Non-augmented (Group 1 & 3) vs Augmented (Group 2 & 4): Participant’s View*

The product information was gathered from the website Discogs. The general information (e.g., label, format, country, released year, genre and style), track list, statistics, companies, and credits information were selected by the researchers. In the conditions without AR, all of the information pages were pasted either physically (Group 1) or virtually (Group 3) onto the edge of the shelf next to each record. In the conditions with AR (Groups 2 and 4), the corresponding information about the LP record was displayed superimposed on the environment based on the image recognition algorithm from the Vuforia Engine. In Group 4, similar to the shop for Group 2 but without using image recognition, the head position of the participant was tracked to predict which record the participant was looking at (see Fig. 2).

#### Apparatus

##### Microsoft HoloLens

In order to superimpose a computer-generated image with the LP record information on the users’ view of the real world and ensure flexible interactions with the products by hand (as in other treatments), the Microsoft HoloLens version 1 AR headset was used for Group 2. Microsoft HoloLens is equipped with a 1280×720 display resolution (per eye) and weighs 579 grams. The digital information only “popped up” whenever the participant was looking directly at the same LP record (front cover) for more than 2 seconds. The straight-line distance between the participant’s head position and the targeted record was between 1 to 1.5 meters.

##### Valve Index

For Group 3 and 4, the participants used the Valve Index headset and its controllers. The Valve Index headset is a fully-immersive headset weighing 809g and is equipped with dual 1440×1600 RGB LCDs, runs at 120Hz, and covers around a 130 degree field-of-view. The Valve Index is accompanied by two controllers (one for each hand) that enable the haptic interface to manipulate records naturally (as in real life) via 87 sensors used to track hand and finger positions.

### Measures

In the present study, the independent variables were composed of the dummy-coded variables of AR (without = 0, with = 1) and VR (without = 0, with = 1). To examine the workload during shopping in extended realities, we employed the NASA-TLX (Task Load Index) (Hart & Staveland, 1988). The NASA-TLX has been used in many empirical studies for subjective workload assessment (see Longo, 2018; Dang et al., 2020). Initially developed at NASA where it was tested and refined in more than forty simulations, it is regarded as a valid and highly reliable source for measuring workload. It has been continually employed and further validated in different contexts for more than thirty years. Due to its high validity, we adopted the NASA-TLX to assess workload, and the procedure is as follows. The first step is to divide each dimension of workload from 0 to 100 in increments of 5. Performance should be given a score between 0 to 100 from good to poor while the other five aspects should be given a score between 0 to 100 from low to high (Hart & Staveland, 1988). Specific to this study, participants were required to rate the six factors of the workload of their shopping separately, namely, *mental demand* (how mentally demanding was the shopping task e.g., thinking, deciding, calculating, remembering, looking, searching?), *physical demand* (how physically demanding was the shopping task e.g., walking, picking, turning, controlling?), *temporal demand* (how hurried or rushed was the pace of the shopping task?), *performance* (how successful were you in accomplishing what you were asked to do in the shopping task?), *effort* (how hard did you have to work mentally and physically to accomplish your level of performance in the shopping task?), and *frustration* (how insecure, discouraged, irritated, stressed and annoyed were you in the shopping task?), after the shopping task was complete. The second step is to obtain a weighted score for each workload factor for calculating the overall workload. Instead of pairwise comparisons, in the current study, participants proceeded to rank the six factors from 0 (least important) to 5 (most important), considering their contribution to the total load of their shopping experience. Thus, for each participant, the weighted score for each dimension was obtained by multiplying the rating score by the corresponding weight. The sum of the weighted ratings was then divided by 15 (the sum of the weights) for an overall workload score.

### Procedures

#### Recruitment

We described the research purpose as “a study of shopping experience” without disclosing any experiment details in the online and offline advertisements posted in a higher education university in Finland. From September to November in 2019, 265 students successfully entered our recruitment system by scanning a QR code or opening the link presented on our advertisement page or flyer (see *Appendix A*). They were then linked to the online self-booking system on Doodle. A total of 165 students participated in the final experiment.

#### Pre-survey

All participants were randomly assigned to join one of the four groups. Before starting the shopping task, researchers first introduced the entire experiment procedure and guided participants to read the consent form for the study. Then, participants filled out a pre-survey related to the relevant information about music products.

#### Tutorial

After the pre-survey, participants got to practice before they started shopping. E*xperimenter 1* guided the participant to the shop room and introduced the experiment procedure step by step, according to the instruction page *(see Appendix B*). For tutorial purposes, the same sample LP record and product information were presented to participants in all shopping conditions.

For Group 2, the experimenters introduced the Microsoft HoloLens and guided participants on how to wear and use the headset to recognize the physical sample LP record displayed outside of the shop room. Two tutorial shopping programs were developed for both Group 3 and Group 4 respectively, without revealing any details of the shops. E*xperimenter 2* was responsible for the tutorial program, and *Experimenter* 1 guided the participant on how to wear a VR headset, move in the virtual space, read information, and interact with the record. The interpupillary distance (IPD) and eye relief for each participant were adjusted carefully during the tutorial.

Each participant was also told that if he/she needed any help or felt uncomfortable during shopping, a short break could be offered during the experiment, and they were welcome to request additional breaks. After ensuring the participants understood the shopping tasks, *Experimenter 1* instructed participants to a) enter the shop room (Group 1 and 2) or b) wait for the program to start (Group 3 and 4), and then start shopping.

#### Shopping task

A pilot study (N = 20) was conducted for all conditions to test the measurement items in the pre-survey and post-survey, as well as the experimental procedure, instruction, shopping program apparatus, and methods. In the actual experiment, participants were asked to spend 10 minutes in the shop and make their purchase decision independently and in accordance with their own preferences. The experimenter played the role of “cashier” in the four shopping conditions. When the shopping time had run out, the “cashier” counted the price of each record product on the check-out table and asked participants to pay with the gift card they had been given. When the shopping was completed and after making sure that the subjects had no physical problems, participants were guided into another room and filled out the post-survey. The 1-3 records that were “bought” by the participants were then restocked with new records of the same price category in the shop.

## Data analyses and results

First, we calculated the overall workload by combining weights and ranks of the sub-dimensions according to the NASA-TLX scale (see section 3.3.1*Measures*). Given that AR (with = 1, without = 0) and VR (with = 1, without = 0) were considered as the two categorical independent variables and overall workload as the (continuous) dependent variable, a between-subjects two-way analysis of variance (ANOVA) was employed. We then conducted a more granular analysis on the effects of AR and VR on each sub-dimension of workload for testing the proposed hypotheses. A two-way multivariate analysis of variance (MANOVA) was conducted to help protect against inflating the Type I error rate in the follow-up two-way analysis of variance (ANOVAs) and post-hoc comparisons.

### Overall workload

The descriptive statistics related to overall workload among the four groups are reported in Table 4 (*N* = 157). It can be seen that the overall workload in the XR-mediated groups (Group 2&3&4) were higher than in the non-XR group (Group1). In addition, Group 2 had the highest overall workload (*M* = 40.081) and Group 1 had the lowest overall workload (*M* = 26.308). In order to test whether AR and VR have an effect on workload, a two-way ANOVA was employed. Even though the normality in one group cannot be assumed according to Shapiro-Wilk results, the two-way ANOVA is reasonably robust to violations of normality when group sizes are similar. Furthermore, the assumption of a homogeneity of variances was tested and satisfied based on Levene’s F test, *F*(3, 153) = 1.576, *p* = .197.

**Table 4:** Descriptive Statistics of Overall Workload in Each Group

Group	VR	AR	*N*	*M*	*SD*	Shapiro-Wilk	
						Statistic	*p*
1	Without	Without	40	26.308	17.689	0.939	.031
2	Without	With	41	40.081	20.861	0.973	.423
3	With	Without	40	34.150	16.076	0.973	.453
4	With	With	36	34.417	15.238	0.964	.288

**Fig. 3:**
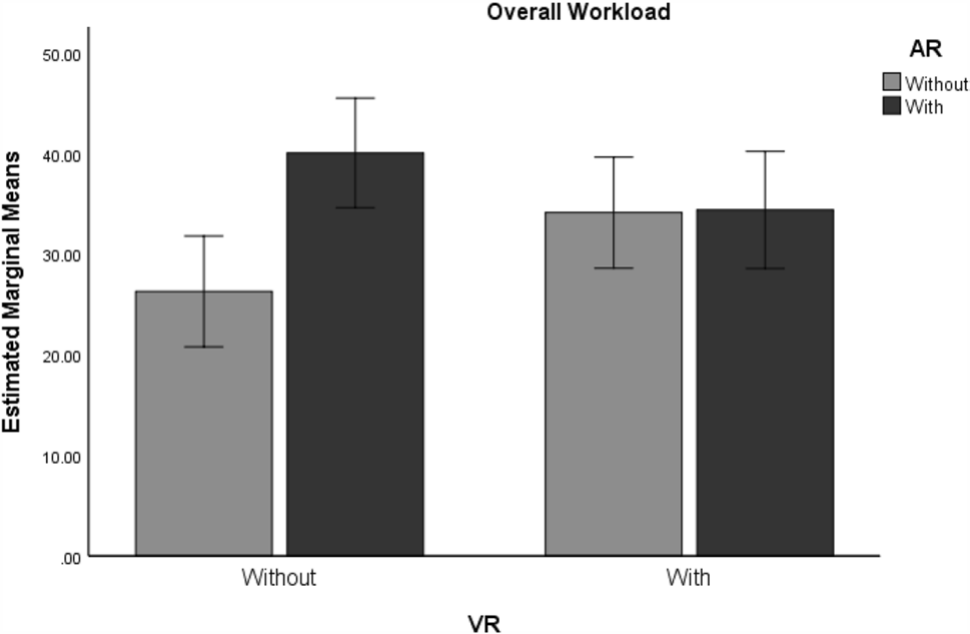
Interaction Effect of AR and VR on Overall Workload

The results reveal that the groups with AR (*M* = 37.249, *SD* = 2.019) had a higher overall workload compared to those groups without AR (*M* = 30.229, *SD* = 1.976, *F*(1, 153) = 6.173, *p* < .05), and AR explains 3.9% of the variance in the dependent variable. On the other hand, the groups with VR (*M* = 34.283, *SD* = 2.031) did not reveal any significantly higher workload in comparison to those groups without VR (*M* = 33.195, *SD* = 1.96, *F*(1, 153) = 0.148 , *p* > .05), and VR only accounts for 0.1% of the variance in workload. The results also show that there was a significant interaction between AR and VR (*F*(1, 153) = 5.713, *p* = .018) and the interaction explains 3.6% of the variance (see Table 5). To interpret the interaction effect, a pairwise comparison was conducted (Bonferroni) using the EMMEANS syntax command within SPSS (see Table 6 and Fig. 3). The results show that only when participants were in the groups without AR, did VR (*M* = 34.150) lead to significantly higher workload than without VR (*M* = 26.308, *p* = .049). Similarly, when participants were in the groups without VR, AR (*M* = 40.081) led to significantly higher workload than those without AR (*M* = 26.308, *p* = .001).

**Table 5:** ANOVA with Overall Workload As Dependent Variable, AR and VR as Independent Variables

Independent variables	*SS*	*df*	*MS*	*F*	*p*	Partial *η*^*2*^
VR	46.389	1	46.389	.148	.701	.001
AR	1929.286	1	1929.286	6.173	.014	.039
VR * AR	1785.492	1	1785.492	5.713	.018	.036

Type III Sum of Squares; SS = Sum of Squares; MS = Mean Square

**Table 6:** Pairwise Comparisons on Overall Workload

	(I)	(J)	*MD* (I-J)	*SE*	*p*	95% *CI*	
						Lower	Upper
Without AR	Without VR	VR	-7.842*	3.953	.049	-15.651	-.032
AR	Without VR	VR	5.665	4.038	.163	-2.312	13.642
Without VR	Without AR	AR	-13.773**	3.929	.001	-21.535	-6.011
VR	Without AR	AR	-.267	4.061	.948	-8.290	7.757

* *p* < .05, ** *p* < .01, *** *p* < .001; MD = Mean Difference; SE = Std. Error

### Sub-dimensions of workload

A series of Pearson correlations were performed among the six dependent variables in each group for testing the MANOVA assumption. The results reveal no concern for multicollinearity, although a few variables were not highly correlated. In addition, the *Box’s M value* of 91.254 was associated with a p-value of .035, which was interpreted as non-significant based on the Hahs-Vaughn (2016) guideline (*p* < .001). The covariance matrices between the groups were assumed to be equal for the purpose of MANOVA. In addition, the absence of multivariate outliers was checked by assessing the Mahalanobis Distances among the participants (the highest value was 22.031 and less than 22.46). A two-way MANOVA was conducted to test the hypothesis that AR and VR have different effects on the various sub-dimensions of workload. A statistically significant MANOVA effect was obtained for the interaction between AR and VR, where Pillai’s Trace = 0.097, *F*(6,148) = 2.643, *p* = .018. The multivariate effect size was estimated at .097, which implies that 9.7% of the variance in the dependent variables was accounted for by the interaction between AR and VR.

#### Hypothesis test

Prior to conducting the ANOVA tests, the homogeneity of variance assumption was tested for all six dependent variables. Based on a series of Levene’s F tests, the homogeneity of variance was considered as satisfied, as all six Levene’s F tests were insignificant (*p* > .05) - see Table 7. A series of two-way ANOVA’s on each of the six dependent variables was conducted as follow-up tests to the MANOVA. As can been seen in Table 7, AR had a significant effect on mental demand (*F*(1, 153) = 5.084, *p* = 0.026, Mean difference = 9.597) and effort (*F*(1, 153) = 6.500, *p* = 0.012, Mean difference = 9.619), while all of the ANOVA’s for VR were statistically insignificant (all *p*-values > .05). Accordingly, regarding the effects of AR on each dimension of workload, *H1a, H4a* and *H5a* were supported, and *H2a, H3a and H6a* were rejected. Regarding the effects of VR on each dimension of workload, *H1b, H3b* and *H5b* were supported while *H2b, H4b* and *H6b* were rejected. In addition, the interaction effect between AR and VR significantly influenced physical demand, *F*(1, 153) = 11.405, *p* =.001 and effort, *F*(1, 153) = 5.647, *p* = .019.

**Table 7:** Two-Way ANOVAs with Workload Subscales As Dependent Variables, AR and VR As Independent Variables

	Levene’s		AR			VR			AR*VR		
	*F*(3, 153)	*p*	*F*(1, 153)	*p*	Partial *η*^*2*^	*F*(1, 153)	*p*	Partial *η*^*2*^	*F*(1, 153)	*p*	Partial *η*^*2*^
Mental demand	0.891	.447	5.084	.026	.032	0.022	.883	.000	0.583	.446	.004
Physical demand	2.562	.057	209.950	.310	.578	0.005	.945	.000	11.405	.001	.069
Temporal demand	1.014	.388	2.264	.134	.015	0.205	.652	.001	2.588	.110	.017
Effort	2.670	.050	6.500	.012	.041	0.139	.709	.001	5.647	.019	.036
Performance	2.567	.057	1.098	.296	.007	0.503	.479	.003	0.469	.495	.003
Frustration	2.087	.104	3.659	.058	.023	0.161	.689	.001	2.029	.156	.013

#### Additional analysis

**Fig. 4:**
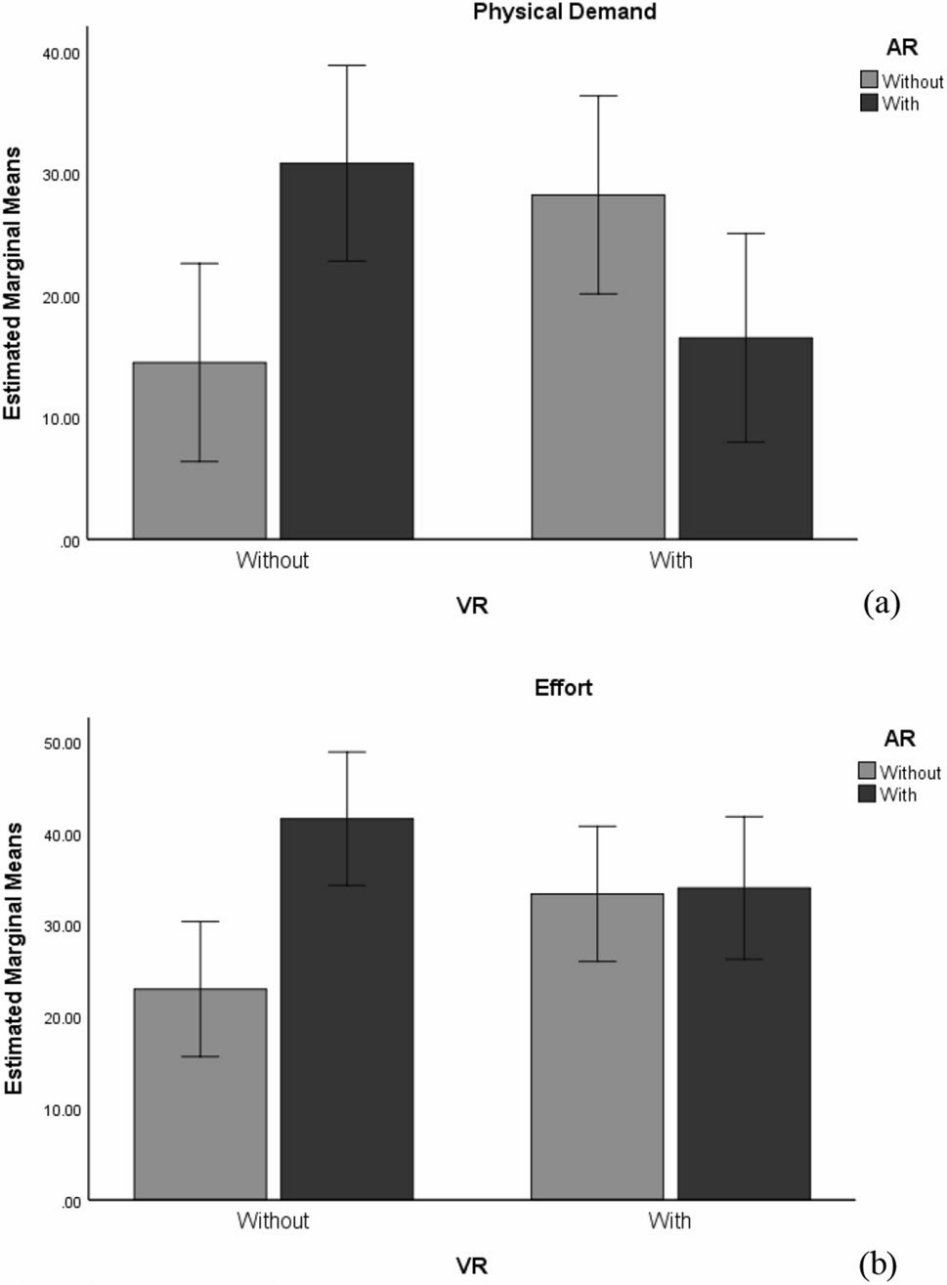
Interaction Effect of AR and VR on Physical Demand and Effort

Finally, a series of post-hoc analyses were performed to examine individual mean difference comparisons across different levels of AR and VR and the two subscales of workload (see Fig. 4 and Table 8). According to the pairwise comparisons (Bonferroni) for groups without AR, VR led to a significant increase in physical demand. In conditions with AR, VR led to a significant decrease in physical demand. Similarly, in the groups without VR, AR significantly led to high physical demand and in the conditions with VR, AR decreased physical demand, albeit insignificantly (*p* = .052). In addition, only in the groups without VR did AR lead to a significant increase in effort compared to those without AR.

**Table 8:** Pairwise Comparisons—Physical Demand and Effort

	(I)	(J)	*MD* (I-J)	*SE*	*p*	95% *CI*	
						Lower	Upper
Subscale: *Physical demand*							
Without AR	Without VR	VR	-13.750*	5.816	.019	-25.240	-2.260
AR	Without VR	VR	14.326*	5.941	.017	2.590	26.062
Without VR	Without AR	AR	-16.354**	5.780	.005	-27.773	-4.934
VR	Without AR	AR	11.722	5.975	.052	-.082	23.527
Subscale: *Effort*							
Without AR	Without VR	VR	-10.375	5.279	.051	-20.804	.054
AR	Without VR	VR	7.558	5.392	.163	-3.095	18.210
Without VR	Without AR	AR	-18.585**	5.247	.001	-28.951	-8.220
VR	Without AR	AR	-.653	5.424	.904	-11.367	10.062

* *p* < .05, ** *p* < .01, *** *p* < .001; MD = Mean Difference; SE = Std. Error

## Discussion

Overall, users experienced higher workload in all three XR-mediated realities than non-XR. To be more specific, AR was significantly associated with overall workload, especially its sub-dimensions of mental demand and effort. In this study, AR was operationalized as “superimposed information” (see Table 2). It is reasonable that when simultaneously processing information from multiple channels and different realities, the cognitive resources users usually spend is higher (Xi et al., 2021). For example, participants in Group 2 (with AR, without VR) had to seek for and process both the digital information presented by the AR headset, and also the information coming from physical products and shopping environment. This high cognitive load was associated with high mental demand and effort.

Additionally, the results also show that using VR did not cause increased perceptions of workload for any of the sub-dimensions, which served to reject the proposed hypotheses that VR has a positive effect on physical demand, effort and frustration. These unexpected results might be explained by the “non-consequentiality” feature of VR. VR has been believed to provide opportunities for a variety of activities that are typically either not done well or safely in real life (Schultheis & Rizzo, 2001; Reid, 2004). Therefore, “non-consequentiality” refers to the feature that the VR-mediated environment could be seen as free of many of the constraints and consequences that govern activities in the real world, and so transforms the environment into playgrounds of free experimentation (Xi & Hamari, 2021). Thus, users can experience a relatively high degree of safety and freedom without concerning any adverse consequences such as physical and psycho-social risks when moving and operating with objects in virtual reality. Such a high degree of freedom without the necessity of concern of consequences might be associated with a low perception of workload. On the other hand, it can also be assumed that there might be yet unmeasured psychological variables or other unexplored features of VR that influence the assessment of difficulties related to using VR for completing certain tasks such as physical demand, effort and frustration.

The interesting findings of the study can be observed in the interaction effects between AR and VR on physical demand, effort and overall workload. Specifically, in terms of physical demand, the combination of AR and VR had a lower physical demand compared to using AR or VR alone. In the present study, the two XR technologies might be associated with physical demand in different aspects: information detection in AR vs. information reading^4^ in VR (low readability - see Peukert et al., 2019); while such negative aspects of AR and VR might be eliminated when the two technologies were combined. One possible reason is that the advantage of AR-based information detection could decrease the difficulty of reading information in virtual reality. In addition, users might have to spend considerable physical resources (head movement, hand movement and general movement) to interact with augmented information as well as content separate in the physical environment, which is not as natural and easy as in the condition combining AR and VR. A similar pattern of interaction effect between AR and VR can be observed on effort and overall workload, and when using AR and VR together, users would not experience higher degrees of effort and overall workload compared with using either VR or AR alone.

## Conclusion

With the development of information technologies, human-computer interfaces and computing power, our daily lives are increasingly lead in technology-mediated environments. Therefore, one of the key research questions is how the workload for carrying out activities as mediated by extended reality technologies may differ both quantitatively and qualitatively from carrying them out without them, as well as how the usability can be improved in the future. Thus, the present study investigated how XR (AR and VR) influence the six dimensions of workload (NASA Task Load Index: mental demand, physical demand, temporal demand, performance, effort, and frustration) and overall workload based on a 2 by 2 between-subject experiment in the retail context. The results from granular analysis indicate that AR was significantly associated with overall workload, especially mental demand and effort, while VR had no significant effect on any of the dimensions of workload. Additionally, the results of the interaction effects show that a combination of AR and VR compared to a single technology would not increase the difficultly of completing tasks (e.g. overall workload and effort), and may even decrease the difficulty (e.g. physical demand). By filling the current gap in research on XR of information systems, human computer interaction and management science, the current study made a considerable research contribution as well as practical guidance for XR designers, developers and practitioners.

### Research implications

The analysis of workload in the present study deepens the understanding of what factors affect usability and efficiency, and what factors are important to users in extended realities, contributing to the corpus of information system and technology-oriented information science. There is a lack of comprehensive understanding and accurate measurement relating to system efficiency and usage barriers in the extant XR-related literature. For a long time, usability has been considered as one of the determinant factors of efficiency in a system (Bangor et al., 2008; Bevan & Macleod, 1994). This construct has mostly been investigated as one single variable in previous studies (system usability score (SUS): see e.g. Sauro, 2011), or measured by perceived ease of use and usefulness under other research frameworks (e.g. the technology acceptance model (TAM): see Davis, 1989). Instead of directly measuring usability as a whole, our study adopted the NASA Task Load Index (NASA-TLX). Even though such a workload-related measure scale is less seen in information systems, it provides strong theoretical support for examining the different dimensions influencing usability when processing information in shopping environments enabled by XR technologies. Beyond offering new insights on how AR and VR independently and interactively influence workload, this study enriches the IS theory by building bridges between fields in the frontier between adoption and ease of use research in relation to new technologies.

In addition, our study provides valuable hands-on research guidance of an experimental design for use in future studies related to the application of XR technologies. We constructed three different XR-mediated shopping environments based on a physical store. Instead of adding interesting interactive interfaces and vivid content in the XR-mediated environment, we aimed at understanding the essence and nature of XR technologies as display methods rather than their information richness (quantity and quality). In order to minimize the potential impact of all possible external factors on the study outcomes, we looked to ensure that all behaviors were to the greatest extent similar in the four shopping conditions by simplifying the shopping programs as close to non-XR shopping conditions as possible, and creating natural interactive ways to shop in each condition. For example, AR was simplified as a technology used to present information (the product information page) in an augmented way. Regarding the devices, given the consideration that consumers needed to interact with products by hand, head-mounted displays were used rather than hand-held devices (e.g., mobile phones or tablets). In addition, we also took the naturalness of interaction into consideration., and instead of using triggers or buttons as controls, two controllers that enabled haptic interfaces for simulating handling products naturally were used in the VR-mediated environments. The rigorous research design aims at ensuring the internal validity of the experiment results.

### Practical implications

The study findings have valuable implications, mainly for XR designers and programmers, and also for practitioners from different areas, especially those in the retail sector. Due to a lack of theoretical guidance and granular analysis of perceived workload, many program developers and designers face the challenge of finding good solutions of how to improve the perceived usability, ease of use, usefulness and efficiency extended towards XR technologies and systems (Gabbard & Swan, 2008; Kaufmann & Dünser, 2007; Shin et al., 2014; Virvou & Katsionis, 2008). The results indicate that the use of AR causes relatively high effort and mental demand compared to conditions without AR. Thus, designers should consider factors such as ease of use, wearability, mobility and interactivity for reducing the required effort in completing tasks with AR, and be more receptive to the degrees of information complexity and richness that might lead to high mental demand, especially when the AR application targets specific groups such as the elderly, children, patients, and the disabled. In terms of VR, similar experiences to those in real life can be emulated without necessarily increasing the difficulty experienced. The results of this study may help reduce the concerns of designers and developers to using VR to create digital experiences that look to replace physical reality. Given that the pure “reconstruction” setting did not increase the difficulty of conducting activities or completing certain tasks, there seems to be an opportunity for adding more vivid content and comprehensive interaction mechanisms into virtual reality, with a relatively low increased challenge of use.

Furthermore, the findings of this study can be applied to retail, and other contexts such as education, training, healthcare, and entertainment. Since the research context is shopping and retail, retail practitioners can directly benefit from the findings of the present study to develop XR retail strategies. One of the main findings is that VR did not increase the challenges for use or offer inferior experiences towards shopping when compared to a situation without VR (e.g., physical reality). Therefore, we encourage retailers to bravely attempt to use VR technology, for example to replace online and offline shopping environments, given that VR can provide a possibility for consumers to shop online at any time and in any place (Bonetti et al., 2018; Rosedale, 2016), and at the same time, immerse consumers in an enhanced artificial reality (such as the 3D environment, a rich interface and natural interactions) that mimics a physical store. Retailers are also encouraged to consider embedding elements of an augmented interface and content into the virtual reality, since AR might bring additional shopping experiences to consumers such as enjoyment, playfulness, fun, etc. (Huang & Liao, 2015; Rese et al., 2017; Spreer & Kallweit, 2014). In addition, practitioners can refer to the findings of the present study and improve the efficiency of XR applications in fields such as learning, working, rehabilitation, and entertainment.

## Limitations

The shopping environment was carefully considered and designed for investigating the matter of perceived workload related to XR, and selected as the research context in the current study. Shopping tasks usually require a lot of physical and cognitive resources, and have been used in many XR-related studies (see e.g. studies on user experience, Speicher et al., 2017; the memory of spatial knowledge, Liang et al., 2019, Man et al., 2011; shopper behavior, Meißner et al., 2019). However, there is still a possibility that VR and AR may have different impacts on the six dimensions of workload in different information systems and application environments. For example, in learning and training, students and trainees usually receive information passively and require more cognitive resources. However, for a technology-related large-scale laboratory experiment, it is unrealistic to design multiple research contexts within a short time. Shopping therefore seems to be the best research context, although we have to admit that one of the limitations of the current study is that the generalization and robustness of the results may not be readily or freely transferable to other sectors.

A second limitation of the research design in our study is the lack of multi-sensory experience in virtual reality, which is associated with user experience. Even though the shops we modeled in virtual reality (Groups 3 and 4) were highly realistic compared to a physical shop, some participants reported that their shopping experiences were a bit “odd” due to the lack of sound and touch experience when moving and interacting with the products. Given that visual experience has been considered as the most important dimension of human sensory experience, we expect that the visual experience itself is sufficient to create the perception of being “there”. Nevertheless, future studies should provide a more multi-dimensional sensory experience in their research design (Xi & Hamari, 2021).

Another limitation is related to the XR devices that were used in the experiments. On one hand, head-mounted displays have limitations in use and are unsuited for certain users. In the current study, two participants were omitted due to a difficulty in wearing / using headsets. Even though the omissions did not influence the results of the experiment, we can still say that there are a certain requirements for XR users which slightly weaken the broad generalization of the findings. On the other hand, two different head-mounted displays were selected to either provide augmenting information in physical reality (Microsoft HoloLens) or to create a virtual reality (Valve Index). Even though participants used these head-based devices and interacted with products in a similar way, there is still a possibility that the statistical differences seen in some areas of the results may be influenced to some degree by the different technical capabilities of the equipment, such as resolution, detection system, field of view, comfort, response time, etc. As pioneering research, the current study made a great effort to investigate comparisons between AR and VR, based on a rigorous experimental method. However, due to relatively irrevocable technical limitations, these potential technical factors might not have been fully controlled in this experiment. Therefore, we encourage future researchers to adopt more improved XR devices to control and reduce any meaningful influence brought by technological difference.

In addition, the underlying mechanism and boundary conditions of how AR and VR influence different dimensions of workload could be investigated by future researchers. For example, our study did not reveal why AR would lead to high effort and mental demand and overall workload (compared to conditions without AR) while VR did not seem to affect any dimensions of workload. Therefore, there might be some important mediating variables (e.g., presence - see Lackey et al., 2016; Lum et al., 2018; flow - see Lackey et al., 2016; emotion - see Cai & Lin, 2011; Lum et al., 2018; Truschzinski et al., 2018) related to the experiential values of AR and VR. More importantly, demographic information was only presented in a descriptive way in the current study, and factors such as gender, age, nationality and income might play moderating roles in differently influencing the difficulty levels of using AR and VR.

As a final consideration, student participants were recruited in the current study for the reason of ready availability, less foreseeable difference within the sample, and an anticipated acceptance towards novel technologies. Therefore, the results might be influenced by the singularity of the sample. Particularly, the younger age groups have been seen to have a higher degree of flexibility, mobility, more tolerance of discomfort, and less difficulty in operating systems and devices. As for most younger users, wearing headsets and using controllers do not seem to restrict their movement and interaction. However, for specific groups such as elder adults, patients, children, or the disabled, there might be more challenges in operating extended realities. Therefore, future studies could investigate the workload of using AR and VR by recruiting participants from other demographic groups.

## Experiment instruction

**Scenario:**

While you are passing by a second-hand LP record shop, you suddenly realize that you have a **10 euro** gift card given by your friend last week. You find out that the expiry date of the gift card is today, which means you have to use it as soon as possible. Thus, you decide to use this gift card to get records for yourself before the shop closes. Remember the shop will close in 10 minutes.

**Gift card:**

This gift card has a 10 euro value. You can use it to buy any records in the shop. Please try to make the best purchasing decision because you can get the records in the end and get them back home. Remember you cannot get any amount of the gift card and you have to use it completely.

**LP record:**

You can pick up records, turn around and read information. Each record has its own price tag on the back. There are **54 records** in the shop in total. Just remember don’t open the cover to avoid scratching the record and each hand can only hold one LP record.

**Extra information:** We provide extra information for each record which can help you to make a better purchase decision. You can find e.g., the artist’s name, album title, released year, style, track-list, company, credit, and social-related information.

**Time:** You need to spend a **full 10 minutes** in the shop. The researcher will knock the door when the timer ends. You are not allowed to use the phone or watch during shopping.

**Purchase decision:** During the shopping time, you can put the selected records on the cashier table and change your selection at any time. We will only ask you to pay the LP records on the cashier table when the time ends. Thus, make sure the total amount of the selected products does not exceed **10 euro**.

**Notice:** If you need any help or feel uncomfortable during shopping, you will be offered to take a short break during the experiment and are welcome to request additional breaks.

## Footnotes

1 The unifying factor of all activities utilizing XR is the use of virtualization in a certain form. Steffen et al. (2019) adapted a previous definition of activities, and referred to these as “virtually assisted activities”.

2 One participant wore a head garment that prevented her wearing a headset, and one participant wore eyeglasses that were incompatible with the VR HMD lenses.

3 Five outliers were identified and dropped for MANOVA and ANOVAs.

4 In this study, *information detection* refers to such features of AR that information available in physical reality such as 2D images (LP record covers) can be detected and tracked, triggering digital content to appear (digital product information page); *information reading* in VR refers to the function of VR that allow users to be able to browse and read digital information.
